# Evidence-based practice usage, knowledge and attitudes of healthcare professionals: a nationwide survey in the Maldives

**DOI:** 10.1136/bmjopen-2024-093609

**Published:** 2025-04-30

**Authors:** Dirk T Ubbink, Aishath Hamid, Fathimath Shifaza

**Affiliations:** 1 Surgery, Amsterdam UMC Location AMC, Amsterdam, The Netherlands; 2 EBP Health Champions Maldives, Hulhumale, Maldives; 3 Australian Catholic University, Ballarat, Victoria, Australia

**Keywords:** Quality in health care, MEDICAL EDUCATION & TRAINING, Nurses

## Abstract

**ABSTRACT:**

**Objectives:**

Evidence-based practice (EBP) is considered an essential principle to arrive at and ensure high-quality healthcare. This study aimed to determine the current knowledge, attitude and awareness among doctors, nurses and allied healthcare workers in the Maldives regarding the principles of EBP and the barriers experienced when practising EBP.

**Design and setting:**

A nationwide, cross-sectional, semiquantitative, digital survey was conducted in 2023 among nurses, doctors and allied healthcare professionals currently working in any healthcare setting in the Maldives. The survey was based on the validated McColl and BARRIERS questionnaires. In addition, basic demographic characteristics of the participants were collected.

**Results:**

Out of the more than 1000 healthcare professionals invited, 418 responded. The vast majority were female nurses. About half of the respondents worked in a tertiary hospital and had obtained a bachelor’s degree in nursing. EBP was considered (very) useful and relevant for clinical practice, but the attitude towards and promotion of EBP was considered insufficient. Respondents preferred research utilisation through evidence-based guidelines. Slightly over half (52.1%) of the respondents had followed some course in literature searching or EBP. Only one in six respondents thought they had access to PubMed. The highest scoring barriers for EBP were related to organisational challenges; lack of knowledge, reluctance to change among healthcare professionals and management, and lack of time or high workload.

**Conclusion:**

Maldivian healthcare professionals welcome EBP but face organisational and practical challenges to implement this principle in clinical practice. A multidisciplinary team of EBP champions appears useful to promote EBP awareness and skills on a national scale.

**STRENGTHS AND LIMITATIONS OF THIS STUDY**

- This was a nationwide survey on the usage, knowledge and attitude towards evidence-based practice (EBP) in the Maldives.
- The survey methods used have been well-tried in other settings and countries.
- More than 1000 out of over 4000 registered healthcare professionals were invited for the survey, of whom about 40% responded, mainly nurses.
- Participants shared their subjective appreciation of EBP, whereas observing EBP in real-time daily practice would be preferable.

## Introduction

 During the last three decades, the paradigm of evidence-based medicine or, as a more general term, evidence-based practice (EBP), has been introduced and promoted by the societal and patients’ demand for professional and resource accountability and improved quality in healthcare.^1 2^ EBP implies that decision-making in healthcare and clinical situations is not only based on the clinical expertise of doctors, nurses and allied healthcare workers but also on the best available evidence from scientific literature, as well as the patient’s situation and preferences.^3^

While EBP is common knowledge in several countries of the North and South American, Australian and European continents, the reality, however, proves that not all healthcare professionals use EBP in their daily practice.^4^,^7^ It has been estimated that in general medicine roughly half of all medical treatments are evidence-based and only about one-fourth of all surgical treatments.^3 8^ Moreover, healthcare professionals report a lack of time, knowledge and basic skills as major barriers to practising EBP.^9 10^

EBP in daily clinical practice requires a joint venture of all healthcare professionals as an interprofessional approach is needed to sustain EBP throughout healthcare systems using the best available evidence. However, healthcare professionals were found to be reluctant towards EBP as they believe research reports are too ‘academic’ and do not offer the desired level of clinical direction when evidence is to be applied to their patients.^11^ Hence, there seems to be room for improvement regarding the implementation of EBP. These improvements in evidence-based behaviour can only be realised and measured, if knowledge, awareness and a positive attitude towards EBP are secured first.^12^ Moreover, strategies to promote change in clinical practice are more likely to be successful if based on a thorough analysis of barriers and facilitators specific to the context.^13^

Although barriers to the use of evidence in clinical practice have been studied worldwide, there are few research reports on this matter addressing the situation in the Maldives.^14^ Healthcare in the Maldives differs from other settings because it is a small, developing country with geographic peculiarities, operating with limited use of technology. The introduction of EBP among all healthcare professionals in the Maldives is being promoted by (non-governmental) organisations such as the EBP Health Champions Maldives,^15^ as well as the Maldivian Nurses Association. In the Maldives, an EBP champion model was introduced in early 2012 and aims to produce clinical leaders from various backgrounds who could implement EBP.^15^ However, the impact of these initiatives is still unclear.

Hence, the aim of this study is to determine the current knowledge, attitude and awareness among doctors, nurses and allied healthcare workers in the Maldives as to the principles of EBP and the barriers experienced when practising EBP. This will help define future tailor-made interventions to improve evidence-based behaviour and thereby the quality of healthcare in the Maldives.

## Methods

This study used a cross-sectional, descriptive design,^16^ consisting of a nationwide, digital, anonymous survey in the Maldives (see online supplemental file 1). The study was reported according to the CROSS checklist.^17^ It was not appropriate to involve patients or the public in the design, or conduct or reporting of our research.

### Participants

Healthcare professionals (doctors, nurses and allied healthcare practitioners) working in any healthcare setting were invited to participate in the survey if they met the following inclusion criteria:

- Qualified healthcare professional working in any healthcare setting in the Maldives.
- Registered with the Ministry of Health, Nursing or Medical Council in the Maldives.
- Involved with direct patient care, or a manager of healthcare professionals involved in direct patient care.
- Holding a current unrestricted licence to practice.

Excluded were:

- University lectures, academics, researchers and students who were not involved in direct patient care.
- Traditional birth attendees and community healthcare personnel.

### Data collection

Data were collected through an online survey. Its content was derived from previous studies and included the McColl and BARRIERS questionnaires,^18 19^ which have been used in several studies before.^6 7 14^ In addition, basic demographic characteristics of the participants were collected.

The McColl questionnaire addresses attitude, awareness and actual usage of EBP. It has been applied ubiquitously.^19^,^21^ In this study, ‘attitude’ was defined as the mind-set of the responders as to the principles of EBP; ‘awareness’ was defined as familiarity with the meaning of certain EBP terms. Also, non-existing dummy terms (‘absolute treatment increase’, ‘dosage chance’, ‘random benefit ratio’, ‘fixed event rate’) were added to detect any socially desirable answering. Respondents answer the seven questions addressing EBP attitude by checking a 10 cm line, ranging from ‘extremely positive’ to ‘extremely negative’ or from ‘fully agree’ to ‘fully disagree’, depending on the topic.

The BARRIERS scale was developed by Funk *et al*^18^ and consists of 29 items. This scale is used to explore the healthcare professionals’ perceptions of barriers to the utilisation of research findings and implementation of EBP in clinical practice. Respondents can score each item as ‘no barrier’, ‘small barrier’, ‘barrier’, ‘large barrier’ or ‘don’t know’. The scale has undergone validation and has been widely used in diverse settings worldwide.^1422^,^25^ In their study, Funk *et al*^18^ categorised the identified barriers into four factors, aligning with dimensions found in Rogers’s diffusion of innovation theory.^26^ The researchers assigned labels to these four factors as follows:

1. Characteristics of the adopter (nurse): eight items.
2. Characteristics of the organisation (setting barriers and limitations): eight items.
3. Characteristics of the innovation (quality of the research): six items.
4. Characteristics of the communication (presentation and accessibility of the research): six items.

The demographic data collected encompassed various participant details, such as age, gender, years of experience, educational qualification and current workplace. Inclusion of the latter item was driven by the organisational structure of the healthcare system in the Maldives, which is interconnected with island-level health centres, atoll-level hospitals, regional hospitals and tertiary hospitals.

The Castor Electronic Data Capturing programme (www.castoredc.com) was used to compile the questionnaire and collect the participants’ responses. Data stored in Castor were only accessible for the study investigators through a login procedure.

Over 1000 eligible participants were invited (by FS) via mass email on 20 January 2023, which contained a link to the digital questionnaire. Email addresses were obtained through the Maldivian Nurses Association, the EBP Champions team and the CEOs of the Maldivian hospitals. Reminders were sent several times until 1 May 2023, after which the database was locked.

### Data management and analysis

Statistical analysis was performed using SPSS V.28 (IBM SPSS, Armonk, New York, USA). Means and SDs were calculated after checking for normal distribution. Student’s t-test is to be used to compare the means of the attitude scores towards EBP between certain subgroups if possible (in particular, medical specialties, doctors vs nurses, males vs females, as these subgroups might differ in their appreciation and application of the EBP-principle). Non-parametric Kruskal-Wallis and Mann-Whitney tests were to be used for non-parametric and subgroup analyses.

Items 1–28 on the BARRIERS scale were loaded into one of the four factors as identified by Funk *et al*.^18^ For each subscale, the mean scores were added. The sum was divided by the number of items in the subscale to attain a comparable figure. ‘Major barriers’ were defined as those scored as a ‘barrier’ or ‘large barrier’. Items for which individuals responded ‘don’t know’ or were left blank were not included in the analysis. No imputation of data was conducted.

In order to identify the largest barriers to research utilisation, the number of respondents who reported each barrier as a major (moderate or large) or minor (no or small) barrier was calculated, and items were rank-ordered accordingly. Descriptive analysis was used for items 30 to 33—other barriers to research utilisation—to identify unique barriers to research utilisation as perceived by the participants. Item 34—the three greatest barriers to nurses’ use of research—was defined as the barriers with the greatest mean scores for the entire sample. Content analysis was used for the open-ended question in item 35-facilitators to research utilisation.

## Results

The survey received responses from 418 participants out of over 1000 healthcare professionals who were invited, resulting in a response rate of approximately 40%. The characteristics of the respondents are detailed in table 1. Respondents had a median of 10 years of experience in patient care. The vast majority of the respondents were female nurses. About half of the respondents worked in a tertiary hospital and had obtained a bachelor’s degree in nursing.

**Table 1:** Respondents’ characteristics

Sex	371 (89.6%) females and 43 (10.4%) males	
Age	Range: 22–65, mean 34.7, median 33 years	
Workplace	Tertiary hospital	201 (51.5%)
	Healthcare centre	92 (23.6%)
	Regional hospital	69 (17.7%)
	Atoll hospital	28 (7.2%)
Current role	Registered nurse	240 (61.5%)
	Clinical nurse	35 (9.0%)
	Enrolled nurse	25 (6.4%)
	Nurse manager	19 (4.9%)
	Lab/cardio technologist	18 (4.6%)
	Registered midwife	15 (3.8%)
	Medical officer	6 (1.5%)
	Doctor	5 (1.3%)
	Pharmacist	4 (1.0%)
	Specialist	3 (0.8%)
	Physical therapist	3 (0.8%)
	Speech pathologist:	1 (0.3%)
	Other	34 (8.7%)*
Highest academic qualification	Bachelor	178 (47.5%)
	Diploma	111 (29.6%)
	Master	56 (14.9%)
	Certificate	25 (6.7%)
	Doctorate	3 (0.8%)
	Other	2 (0.5%)†
Experience in patient care	Range: 0–50, mean 11.2, median 10 years	
Administrative region	Upper North	43 (11.5%)
	North	14 (3.7%)
	North Central	12 (3.2%)
	Central	134 (35.7%)
	South Central	10 (2.7%)
	South	162 (43.2%)

* Attendant: 4%, radiographer: 3%, primary healthcare worker: 1%, professor: 1%.

† Advanced nursing.

### McColl questionnaire results

Concerning the attitude towards EBP among the respondents, the highest level of agreement was observed regarding the usefulness of EBP in enhancing patient care. Conversely, the current attitude towards, and promotion of, EBP was deemed insufficient by the respondents. Further details are shown in table 2.

**Table 2:** Respondents’ attitude towards EBP

Question	Mean score (SD)	N
Opinion about current EBP promotion	65.7 (23.8)*	307
EBP attitude of colleagues	63.7 (22.1)*	298
Usefulness of research findings in practice	81.0 (19.2)*	289
Percentage of practice that is evidence-based	71.4 (21.2)	286
Practising EBP improves patient care	88.6 (15.7)*	282
EBP less useful: many interventions lack evidence	60.6 (23.5)*	258
Adopting EBP is too demanding for busy professionals	70.2 (21.9)*	272

* 0=disagree/worst, 100=agree/best.

EBP, evidence-based practice.

For the purpose of EBP, most respondents preferred evidence-based guidelines or protocols, both now and in the future (table 3).

**Table 3:** Respondents’ preferred evidence-based practice

	Using now	Like to use	Most suitable
Learning EBP skills	107 (25.6%)	136 (32.5%)	106 (25.4%)
Searching EBP summaries	94 (22.5%)	130 (31.1%)	109 (26.1%)
EB guidelines/protocols	171 (40.9%)	171 (40.9%)	166 (39.7%)

EBP, evidence-based practice.

About half (52.1%) of the respondents had followed a course in searching the literature, while one-third had attended a training in critical appraisal (36.0%) or an EBP course (37.7%). Participants had conducted a median of two literature searches in the past month.

**Figure 1:**
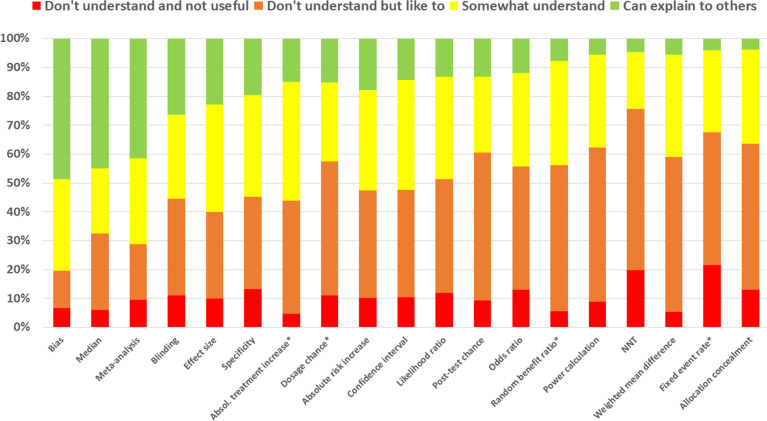
Knowledge of evidence-based practice terms. Asterisks indicate dummy terms.NNT, Number Needed to Treat.

Although one-third (32.8%) of the respondents had internet access at home and a quarter (25.1%) at work or school, access to PubMed was stated in 16% and 15.8%, respectively. Apparently, respondents were unaware that PubMed is freely accessible for anyone with internet access. Among the sources of EBP, respondents showed the highest level of awareness for *Evidence-Based Nursing Journal*, the CINAHL database and the Cochrane database. The knowledge of EBP terms is shown in figure 1. Dummy terms are indicated by an asterisk.

### BARRIERS questionnaire results

Table 4 shows the mean score (range: 0–3) with SD of each barrier, as well as the percentage of respondents who considered this a major barrier. Highlighted are the five highest scoring major barriers (scores above 66%). All except one were related to organisational challenges.

**Table 4:** BARRIERS scale items

	Mean	SD	Perceived as major barrier (%)
Adopter characteristics			
The nurse does not see the value of research for practice	1.53	0.99	48.6
The nurse sees little benefit for self	1.59	0.96	56.1
The nurse is unwilling to change/try new ideas	1.90	0.92	61.5
There is not a documented need to change practice	1.63	0.93	57.7
The nurse feels the benefits of changing practice will be minimal	1.85	0.87	65.3
The nurse does not feel capable of evaluating the quality of the research	1.56	0.90	50.0
The nurse is isolated from knowledgeable colleagues with whom to discuss the research	1.48	0.98	58.2
The nurse is unaware of the research	1.81	1.00	63.5
Organisation characteristics			
Administration will not allow implementation	1.71	1.05	55.7
Physicians will not cooperate with implementation	1.88	0.85	66.7
There is insufficient time on the job to implement new ideas	1.82	0.98	64.4
Other staff are not supportive of implementation	1.71	0.95	56.2
The facilities are inadequate for implementation	1.95	0.92	72.9
The nurse does not feel she/he has enough authority to change patient care procedures	2.00	0.88	72.9
The nurse does not have time to read research	1.99	0.94	68.3
The nurse feels results are not generalisable to own setting	1.80	0.95	63.8
Innovation characteristics			
The research has methodological inadequacies	1.63	0.86	60.6
The conclusions drawn from the research are not justified	1.55	0.91	51.5
The research has not been replicated	1.76	0.95	62.1
The literature reports conflicting results	1.53	0.86	50.0
The nurse is uncertain whether to believe the results of the research	1.67	0.83	58.6
Research reports/articles are not published fast enough	1.53	0.97	57.3
Communication characteristics			
Implications for practice are not made clear	1.62	0.83	56.5
Research reports/articles are not readily available	1.84	0.91	63.4
The research is not reported clearly and readably	1.55	1.03	52.3
Statistical analyses are not understandable	1.87	0.93	66.3
The relevant literature is not compiled in one place	1.76	0.95	63.3
The research is not relevant to the nurse’s practice	1.39	1.08	46.8

Most frequently reported items are highlighted.

When asked what the three most important barriers are regarding implementation of EBP, the respondents chose a lack of knowledge, a reluctance to change among healthcare professionals and management, and a lack of time or high workload. The most frequently mentioned facilitators for EBP were skills, training, awareness and time.

## Discussion

This national digital survey among Maldivian healthcare professionals showed a positive attitude towards EBP as the respondents considered the utilisation of research findings essential to improve the quality of patient care. For this purpose, evidence-based guidelines and protocols are preferred. More than half of Maldivian healthcare professionals had followed some course related to EBP, but no structural training as part of their professional education. The majority of the Maldivian healthcare professionals still need and desire training to be able to apply EBP in clinical practice, which calls for the incorporation of EBP in the educational curricula. On the other hand, time restraints, a lack of skills and a reluctance to change habits are hampering the implementation of EBP. This suggests that also the management of healthcare organisations should make more room for EBP activities among their personnel, realising this is an important means to improve quality of care.

These overall results show there has been little progress since an earlier inventory in 2014 of EBP barriers among Maldivian nurses.^14^ Nurses in particular tend to feel hierarchically subordinated to doctors and may therefore feel their initiatives to change are thwarted by ‘unwilling’ physicians or managers. An interprofessional approach, in which every healthcare professional is allowed—and even stimulated—to have a positive but critical attitude towards why things are done the way they are done in current practice, may lead to a concerted focus on evidence-based discussions and improvements.

The promotion of EBP has been a challenge to many countries and healthcare systems. A multifaceted approach on various levels is advocated to involve healthcare professionals, managers and policymakers in the implementation of EBP in the healthcare system.^27^,^29^ This includes improving knowledge and skills among healthcare professionals, promotion of the paradigm by the executive officers, a culture conducive to EBP, facilitating access to digital sources of evidence, allowing dedicated time to do literature searches, critical appraisals of scientific publications and evidence-based updates of guidelines and protocols based on best available evidence. Education of future doctors and nurses by paying structural attention to EBP in their curricula will produce a next generation of healthcare professionals whose focus is set on applying this principle in their daily healthcare activities and who will be role models of how to practice EBP.^30^ To improve and maintain EBP skills among practising healthcare professionals, one could consider publishing a (yearly) inventory of critically appraised topics, evidence-based updated protocols and regular EBP trainings and journal clubs.^31 32^ A group of EBP champions, who have received specific EBP education to foster its implementation, is likely essential to boldly proclaim and teach the values and skills of EBP among their peers.^15^ A recent systematic review showed that the presence of champions or role models may lead to increased use of EBPs, programmes or technological innovations at the organisational level.^33^

An overview of practical suggestions to improve EBP, based on this survey, is shown in the below.

### How to improve EBP by healthcare professionals, directors and policymakers

- Create more awareness and acceptance of the EBP paradigm as a means to improve quality of care among all healthcare professionals.
- Stimulate EBP by structural incorporation of EBP education in medical and nursing curricula.
- Allow current healthcare professionals to enhance their EBP skills through regular EBP courses and workshops, preferably fostered by a dedicated team of EBP champions.
- Dedicate time for literature search and critical appraisal of recent evidence.
- Produce and update local protocols and existing guidelines based on recent evidence.
- Maintain your EBP skills, for example, through regular journal clubs.

### Study limitations

According to the National Bureau of Statistics, there are some 1200 doctors, 3000 nurses and 600 allied health staff registered in the Maldives,^34^ while more than 1000 eligible healthcare professionals were invited for the survey. This means a relatively small sample was eventually captured in this survey. This may be due to time restraints of busy healthcare professionals or insufficient promotion of this study. The final number of respondents limited our possibilities of doing subgroup analyses of medical specialties, nurses versus doctors and males versus females. Still, it is one of the largest national surveys on EBP that have been conducted so far.

As this study aimed to sample all types of healthcare professionals in the Maldives, it is likely to be representative for, and generalisable to, the national situation in the Maldives, but also for any country that desires to implement EBP in their healthcare system, because of the generic character of the EBP principle. Although the survey comprised a small proportion of doctors, the perceived barriers towards EBP, like lack of time, training opportunities and organisational challenges, are generic issues that affect any healthcare professional. Because the survey mostly captured nurses, generally being the largest workforce in healthcare, the survey is particularly informative about their appreciation of EBP.

Another limitation of this study may be that the attitude, awareness and barriers towards EBP were assessed subjectively, that is, as perceived by the participants themselves. One would rather determine these parameters during ‘real-time’ daily practice. However, valid assessment instruments to evaluate EBP behaviour are still lacking.^35^ One could consider installing a (yearly) inventory of critically appraised topics, evidence-based updated protocols, and regular trainings and journal clubs to maintain and improve EBP skills.^31^

## Supplementary material

10.1136/bmjopen-2024-093609 online supplemental file 1

## Data Availability

Data are available upon reasonable request.
