# Supplementary material for: Evidence-based practice usage, knowledge and attitudes of healthcare professionals: a nationwide survey in the Maldives
Source: BMJ Open. 2025 Apr 30;15(4):e093609. doi: 10.1136/bmjopen-2024-093609 (PMC12049935; doi:10.1136/bmjopen-2024-093609)
Supplement: online supplemental file 1 [file bmjopen-15-4-s001.docx]

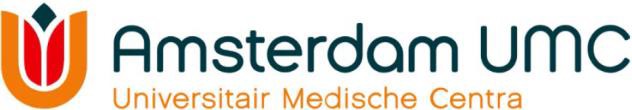

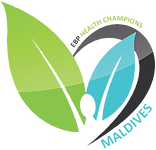


## **Baseline evidence-based practice use, knowledge, and attitudes of healthcare professionals in the Maldives**

Dear Healthcare practitioner,

We are conducting research that focuses on translating evidence-based practice in the Maldives. Evidence-based practice is an approach that has gained recognition for facilitating the transfer of evidence into quality clinical practice.

The purpose of this questionnaire is to measure the baseline evidence-based practice use, knowledge, and attitudes among healthcare professionals in the Maldives. It is NOT a test or an evaluation of you as a clinician. It is anonymous. As a clinician, you are invited to take part in this research project. There are 2 parts to this questionnaire. Part A consists of demographic data and Research Utilization Questionnaire and Part C contain 29 items of the BARRIER scale. It will take ten to fifteen minutes to fill in this questionnaire. By completing this questionnaire, we assume that you have consented to be part of this research. The success of this research depends on the generosity of your participation to spend time as a participant.

Thank you for sharing your views with us

Yours sincerely,

**Research Team:**

Prof. Dirk T. Ubbink, MD PhD MSc, Professor in Evidence-based medicine and Shared decision-making. Amsterdam University Medical Centers at the University of Amsterdam, location AMC, Department of Surgery, Meibergdreef 9, 1105AZ, Amsterdam, The Netherlands.

Ms. Aishath Hamid, RN, RM, MSc EBP Health Champions Maldives, 41-2-5 Nirolhu Magu, Hulhumalé, 23000, Maldives.

Ms Aminath Zeeniya, RN, RM, Msc. General Manager, Aasandha Limited, Maldives

Ms

Ms Mariyam Zulfa, RN, RM, MSc. Associate Director of Nursing Practice, Addu Equitorial Hospital, Maldives

Ms Mariyam Huda, RN, BScN, MBA. Nursing Director, ADK Hospital, Maldives.

Ms Thasleema Abdul Raheem, RN, RM, BSc, Finey Health centre, Maldives

Dr. Fathimath Shifaza, RN, RM MSc, PhD, FACN**,** Senior Lecturer Nursing, Post graduate Course Coordinator, Flinders University, South Australia

Respondent number

**General Information**

Please check ﴾√ ﴿ the correct answer for the questions below:

1. **Gender:**

□ male □ female

1. **Age**

1. **Highest Academic Qualification ( Please tick and complete)**
   - Certificate in ………….
   - Diploma in …………………..
   - Bachelor’s degree in …………………
   - Master’s degree in …………..
   - Doctorate degree in ……………..
   - Other ( Please specify)………………
2. **Which of the following best describes your current role**
   - Doctor
   - Superspecialist,
   - Specialist,
   - Medical officer
   - GP
   - Registered Nurse
   - Clinical Nurse
   - Nurse Manager
   - Enrolled Nurse
   - Registered Midwife
   - Physiotherapist
   - Speech pathologist
   - Pharmacists
   - Dentists
   - Other registered health professions (please specify)…………..

**Years of experience in patient care:**

Years

1. **Current workplace**
   - Healthcare Centre
   - Atoll Hospital
   - Regional Hospital
   - Tertiary Hospital
2. **Administrative Region**
   - Upper North Region ( Haa Alif, Haa Dhaalu, Shaviyani and Noonu Atoll)
   - North Region ( Raa, Baa, Lhaviyani and Kaaf)
   - North Central Region ( Alif Alif, Alif dhaalu, Vaavu, Meemu and Faafu)
   - Central Region ( Male’ Region)
   - South Central region ( Dhaalu, Thaa and Laam)
   - South Region ( Gaaf Alif, Gaaf Dhaal, Gnaviyani and Seenu)

PART I: Ideas among Healthcare Professions about EBP

*Evidence-based practice (EBP) has become a popular term and is one of the pinnacles of modern healthcare. The EBP principle encourages the use of the best available evidence from scientific literature in combination with clinical experience and the patient’s preference.*

*We want to study to which extent the EBP principle is known in the Healthcare profession and is integrated into clinical practice. We also want to know what the perceived facilitators and barriers are. This will help promote EBP and improve the quality of care for our patients.*

*Now we would like to ask you about your opinions regarding EBP. Check the line at the point that agrees with your opinion or answer, for example:*

EXAMPLE: “The world is threatened by mass nuclear destruction”

X

Fully agree Fully disagree

1. **What is your opinion about the way EBP is currently promoted?**


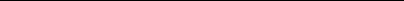


Extremely positive Extremely negative

1. **How would you characterize the attitude of the majority of your direct colleagues towards EBP?**


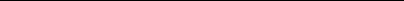


Extremely positive Extremely negative

1. **How useful are research findings in your daily care for patients?**


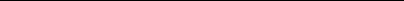


Extremely useful Completely useless

1. **Which percentage of your clinical practice do you think is evidence-based?**


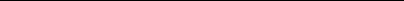


100% 0%

1. **Practicing EBP improves patient care.**


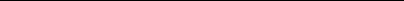


Fully agree Fully disagree

1. **EBP is of limited value in clinical practice because many interventions lack scientific support.**


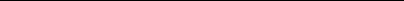


Fully agree Fully disagree

1. **Adopting EBP is a valuable idea but is demanding for already very busy nurses.**


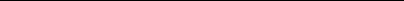


Fully agree Fully disagree

*A clinician can move from practising based on opinions and experience towards practising based on evidence:*

1. By learning EBP skills; searching and critically appraising original publications or systematic reviews.
2. By searching and applying evidence-based summaries that provide the clinical key messages. These can be obtained from Journals with these summaries.
3. By using evidence-based practice guidelines or protocols developed by colleagues.
4. **Which of these methods do you use now?**

(You may check more than one box) a
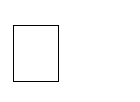
 b
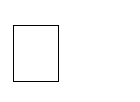
 c
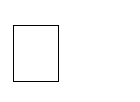


1. **Which of these methods would you like to use in the future?**

(You may check more than one box) a
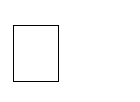
 b
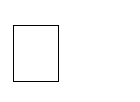
 c
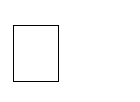


1. **Which of these methods do you think is most suited for your profession?**

(You may check more than one box) a
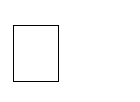
 b
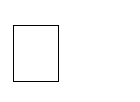
 c
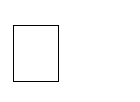


1. **Can you describe how EBP can be facilitated in clinical practice?**
2. **How often have you (or someone on your behalf) searched PubMed or another bibliographic database to search for literature?**

times

18a. When was the last time you did a literature search that influenced your practice?

Month: Year:

18b. **What was the topic?**

19a. Did you ever receive formal training in doing a literature search?

Yes
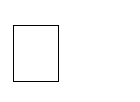
 No
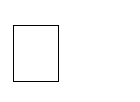


19b. **If yes, where?**

20a. Did you ever receive formal training in critically appraising literature?

Yes
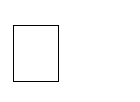
 No
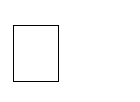


20b. **If yes, where?**

21a. Did you ever follow a course in EBP?

Yes
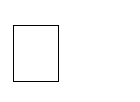
 No
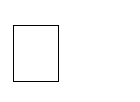


21b. **If yes, where?**

1. **Where do you have access to PubMed (or another bibliographic database)?**

22a. **At home**

Yes
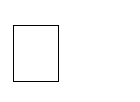
 No
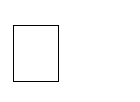


22b. At my department/ward

Yes
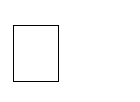
 No
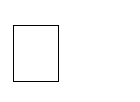


22c. In my local (medical) library

Yes
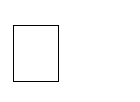
 No
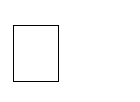


22d. **Somewhere else:**

1. **Where do you have access to the internet?**

23a. **At home**

Yes
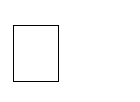
 No
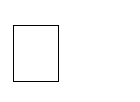


23b. At my department/ward

Yes
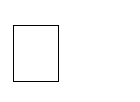
 No
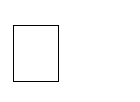


23c. In my local (medical) library

Yes
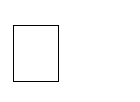
 No
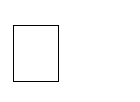


23d. **Somewhere else:**

*A growing number of sources offer summaries relevant to EBP. Please indicate which of these you use or know about.*

|  | **Don’t know it** | **Know it, but haven’t read it** | **Have read it** | **Used it for clinical decision-making** |
| --- | --- | --- | --- | --- |
| Evidence-Based Nursing | □ | □ | □ | □ |
| JBI Evidence Implementation | □ | □ | □ | □ |
| CINAHL | □ | □ | □ | □ |
| Cochrane Database | □ | □ | □ | □ |
| Word Views on Evidence-based Nursing | □ | □ | □ | □ |
| Journal of Evidence-Based Medicine | □ | □ | □ | □ |

*The following terms are used in EBP articles and may be relevant to your profession. Please indicate to which extent you understand each term.*

|  | **I don’t understand the term and I don’t think it is useful for me to understand it.** | **I don’t understand the term, but I would like to understand it.** | **I somewhat understand the term.** | **I understand the term and could explain it to others.** |
| --- | --- | --- | --- | --- |
| Absolute treatment increase | □ | □ | □ | □ |
| Bias | □ | □ | □ | □ |
| Blinding | □ | □ | □ | □ |
| Dosage chance | □ | □ | □ | □ |
| Effect size | □ | □ | □ | □ |
| Median | □ | □ | □ | □ |
| Meta-analysis | □ | □ | □ | □ |
| N.N.T. | □ | □ | □ | □ |
| Power calculation | □ | □ | □ | □ |
| Specificity | □ | □ | □ | □ |

Part II Perceived barriers to EBP

*We would like to know to which extent you think the following statements are a barrier for nurses to use research findings. For each statement, circle the number that best matches your opinion.*

*
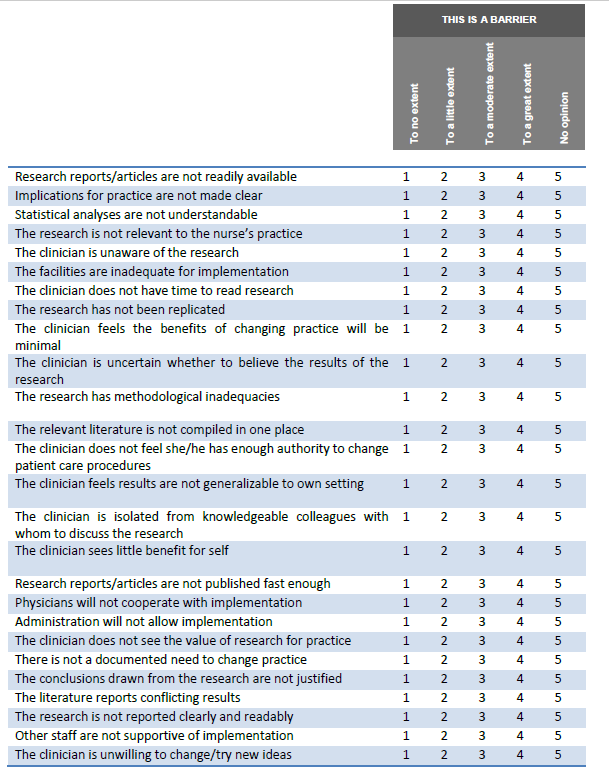

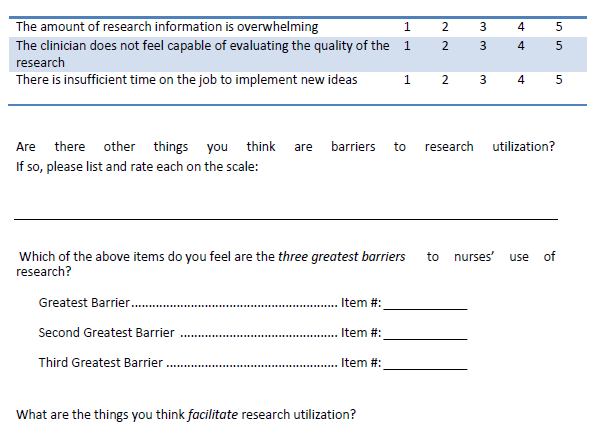
*
