# Supplementary material for: Evidence-based practice usage, knowledge and attitudes of healthcare professionals: a nationwide survey in the Maldives
Source: BMJ Open. 2025 Apr 30;15(4):e093609. doi: 10.1136/bmjopen-2024-093609 (PMC12049935; doi:10.1136/bmjopen-2024-093609)
Supplement: Supplementary file 2 [file bmjopen-2024-093609.reviewer_comments.pdf]

PEER REVIEW HISTORY

BMJ Open publishes all reviews undertaken for accepted manuscripts. Reviewers are asked to complete a checklist review form and are provided with free text boxes to elaborate on their assessment. These free text comments are reproduced below.

ARTICLE DETAILS

Title (Provisional)

Evidence-based practice usage, knowledge, and attitudes of healthcare professionals: A nationwide survey in the Maldives

Authors

Ubbink, Dirk T.; Hamid, Aishath; Shifaza, Fathimath

VERSION 1 - REVIEW

**Reviewer**	**1**
**Name**	**Kulmala, Petri**
**Affiliation**	**University of Oulu, Medical Faculty**
**Date**	**15-Nov-2024**
**COI**	**None**

This research focuses on a scientifically relevant and timely issue closely related to the quality of health care and competence of health care professionals, in the light of evidence-based practice. Although this study is descriptive and scoping in nature, it provides a valuable set of information in the context of a developing, low-income country. For me the manuscript looks to be carefully prepared and written in fluent English that was easy to follow. The tools selected for data collection and analyses were appropriate, reliable (e.g. validated questionnaires) and well described. I have only a few concerns to be noted:

- As stated in the Study Limitations -section, the study sample is not optimal in size, especially when the distribution of the respondents is taken into account. A reasonable size of 1,000 health care professionals was chosen as a sample to be enrolled to this study. 40% response rate is fairly common in surveys and in such does not invalidate the significance of the observations found in this study. However, the distribution/proportions (or any estimation of) of various health care personnel among these 1,000 professionals was not described, which makes the evaluation of the representativeness of the sample and interpretation of the results and validity more challenging

- The very low number of other respondents (especially doctors) than nurses is a major concern (ref Table 1). This has a direct impact on the interpretation and generalizability of the results. Now, along with the entire manuscript, authors state and generalize the results

under the term “health care professionals”, despite the fact, that the results are undoubtedly not representative for e.g. doctors (1.3% of the respondents of this study and 0.4% (5/1200) of the total number of doctors in Maldives). The statements and interpretations proposed in the manuscript should be made with caution, taken into account these facts.

**Reviewer**	**2**
**Name**	**Santos, D**
**Affiliation**	**Universidade de Coimbra**
**Date**	**07-Mar-2025**
**COI**	**None**

It was a pleasure to have the opportunity to review your paper.

In my opinion the article is well structured and adds very interesting knowledge to the scientific evidence.

From the review, I only have the following suggestions/questions:

#1 Are the instruments used to collect data validated for the population used?

#2 How did you get access to the participants?

#3 If it were possible, the wording of the results would, in my opinion, be worthy of more in-depth study. For example, avoid starting with "The attitude towards EBP among the respondents is shown in Table 2". I think it would be more useful for readers to first receive a description of the results and only then a mention of the table.

**Reviewer**	**3**
**Name**	**Jongerden, Irene P.**
**Affiliation**	**Vrije Universiteit Amsterdam**
**Date**	**12-Mar-2025**
**COI**	**None**

Thank you for the opportunity to review this well written manuscript, entitled “Evidence-based practice usage, knowledge, and attitudes of healthcare professionals: A nationwide survey in the Maldives”. The manuscript addresses the ongoing important topic of applying evidence based practice by health care professionals in a developing country. Through a survey (n=418, mainly nurses), they explored the current knowledge, attitude and awareness as well as barriers regarding EBP.

The introduction is clear and referring to relevant literature, though not extensive given the many studies that have been conducted related to EBP, barriers and strategies. One of these strategies is the introduction of EBP champions, often locally, and in the Maldives promoted nationally. The authors state that the impact of these initiatives is still unclear (line 84), and subsequently focus on aims that address knowledge, attitude and barriers. Explaining more about the initiatives (in the introduction or methods section) and elaborate or reflect upon their probable impact (in the discussion section) would add to the additional value of the manuscript, especially since the authors mention little progress since a previous inventory in 2014 (lines 217-218).

Related, practical suggestions (lines 239 and on) are results, should have been mentioned somehow in the results section. Did these suggestions come from open ended questions? Or do they represent the authors suggestions based on the results?

Related, I was wondering to what extend EBP is embedded in educational programs of HCP. One of the practical solutions suggests that EBP is yet not embedded, is that a correct observation?

In the methods section, the use of two questionnaires is mentioned. To me it was not clear whether the used definitions were in line with the original questionnaires (lines 117-119 and 128-129). In addition, I would prefer more information about the total number of questions and whether answers were scored dichotomous, Likert scale or continuous. Related, I would recommend to motivate the possible subgroup analysis in advance: what groups did you want to compare (not 'for example') and why?

Related, I was wondering whether >1.000 (exact number?) out of almost 5.000 HCP in the Maldives are registered with the Ministry, or a selection was made when inviting HCP to participate

Related, please provide IQR or SD in the tables where medians and means are provided.

The largest group of respondents were nurses (total >80% RN, CNS, enrolled nurse, nurse manager). These professionals often perceive more barriers in using EBP. Can you elaborate on the effect on outcomes in your study (i.e. more positive due to other HCP, or less progress due to this large group of nurses). Related, it was not always clear to me whether HCP in general or nurses, physicians or clinicians were referred to (i.e. line 130, line 164 and some of the questions: nurse or HCP?).

In the abstract, a semi quantitative design is mentioned, and in methods section a descriptive design. The latter seems more appropriate.

VERSION 1 - AUTHOR RESPONSE

Replies to reviewer 1

Prof. Petri Kulmala, University of Oulu, Oulu University Hospital

Comments to the Author:

This research focuses on a scientifically relevant and timely issue closely related to the quality of health care and competence of health care professionals, in the light of evidence-based practice. Although this study is descriptive and scoping in nature, it provides a valuable set of information in the context of a developing, low-income country. For me the manuscript looks to be carefully prepared and written in fluent English that was easy to follow. The tools selected for data collection and analyses were appropriate, reliable (e.g. validated questionnaires) and well described. I have only a few concerns to be noted:

- As stated in the Study Limitations -section, the study sample is not optimal in size, especially when the distribution of the respondents is taken into account. A reasonable size of 1,000 health care professionals was chosen as a sample to be enrolled to this study. 40% response rate is fairly common in surveys and in such does not invalidate the significance of the observations found in this study. However, the distribution/proportions (or any estimation of) of various health care personnel among these 1,000 professionals was not described, which makes the evaluation of the representativeness of the sample and interpretation of the results and validity more challenging

Answer: The invitation to participate in this survey was sent out to more than 1,000 healthcare professionals in a commensurate manner, so the response rates were likely to be similar among doctors and nurses. As we do not have these exact numbers, we did not specify this in the manuscript. We have added extra information on the invitation procedure in lines 153-156.

- The very low number of other respondents (especially doctors) than nurses is a major concern (ref Table 1). This has a direct impact on the interpretation and generalizability of the results. Now, along with the entire manuscript, authors state and generalize the results under the term "health care professionals", despite the fact, that the results are undoubtedly not representative for e.g. doctors (1.3% of the respondents of this study and 0.4% (5/1200) of the total number of doctors in Maldives). The statements and interpretations proposed in the manuscript should be made with caution, taken into account these facts.

Answer: We fully agree with the reviewer that the proportion of 'non-nurses' in the survey is below expectation. On the other hand, the perceived barriers towards EBP, like lack of time, training opportunities, and organizational challenges, are generic issues that affect any healthcare professional. We have added this notion in lines 281-283.

Replies to reviewer 2
Dr. D Santos, Universidade de Coimbra
Comments to the Author:
It was a pleasure to have the opportunity to review your paper.
In my opinion the article is well structured and adds very interesting knowledge to the scientific evidence.
From the review, I only have the following suggestions/questions:
#1 Are the instruments used to collect data validated for the population used?

Answer: The questionnaires used in this survey have indeed been validated and well-tried in previous studies among general practitioners, clinical nurses, residents and medical specialists. See reference numbers 6, 7, 14, 18-25.

#2 How did you get access to the participants?

Answer: Participants were accessed through mass email by FS. Their addresses were obtained through the CEOs of the Maldivian hospitals, the Maldivian EBP Champions network, and the Maldivian Nurses Association, in order to reach as many healthcare workers as possible. Also, the survey was promoted by word of mouth. This is now reported in lines 154-156.

#3 If it were possible, the wording of the results would, in my opinion, be worthy of more in-depth study. For example, avoid starting with "The attitude towards EBP among the respondents is shown in Table 2". I think it would be more useful for readers to first receive a description of the results and only then a mention of the table.

Answer: Thank you for this remark. We have now added more information regarding the results in the text before referring to the tables (lines 191-194).

Replies to reviewer 3
Dr. Irene P. Jongerden, Vrije Universiteit Amsterdam
Comments to the Author:
Thank you for the opportunity to review this well written manuscript, entitled "Evidence-based practice usage, knowledge, and attitudes of healthcare professionals: A nationwide survey in the Maldives". The manuscript addresses the ongoing important topic of applying evidence based practice by health care professionals in a developing country. Through a survey (n=418, mainly nurses), they explored the current knowledge, attitude and awareness as well as barriers regarding EBP.

The introduction is clear and referring to relevant literature, though not extensive given the many studies that have been conducted related to EBP, barriers and strategies. One of these strategies is the introduction of EBP champions, often locally, and in the Maldives promoted nationally. The authors state that the impact of these initiatives is still unclear (line 84), and subsequently focus on aims that address knowledge, attitude and barriers. Explaining more about the initiatives (in the introduction or methods section) and elaborate or reflect upon their probable impact (in the discussion section) would add to the additional value of the manuscript, especially since the authors mention little progress since a previous inventory in 2014 (lines 217-218).

Answer: Thank you for this remark. We have now elaborated on the effect of EBP Champions and have added a reference to another study on this in the Discussion section (lines 251-254).

Related, practical suggestions (lines 239 and on) are results, should have been mentioned somehow in the results section. Did these suggestions come from open ended questions? Or do they represent the authors suggestions based on the results?

Answer: The authors derived these practical suggestions from the results of this survey and were not direct results of questions included in the survey. That is why these were placed in a frame in the Discussion section.

Related, I was wondering to what extend EBP is embedded in educational programs of HCP. One of the practical solutions suggests that EBP is yet not embedded, is that a correct observation?

Answer: That is correct; educational programs have not (yet) been structurally implemented in the Maldives. In the Discussion we indicated that more than half of Maldivian healthcare professionals had followed some course related to EBP, but no structural training as part of their professional education. The EBP Champions team does have introduced Journal Clubs to critically appraise relevant publications.

In the methods section, the use of two questionnaires is mentioned. To me it was not clear whether the used definitions were in line with the original questionnaires (lines 117-119 and 128-129). In addition, I would prefer more information about the total number of questions and whether answers were scored dichotomous, Likert scale or continuous. Related, I would recommend to motivate the possible subgroup analysis in advance: what groups did you want to compare (not 'for example') and why?

Answer: These definitions of 'attitude' and 'awareness' were provided to specify their meaning for the reader and did not deviate from the original questionnaire.

We did not further elaborate on the questionnaires, being well-known and frequently used. Nevertheless, the manuscript now contains brief additional information about the answering formats in these questionnaires (lines 129-131 and 134-135).

The McColl questionnaire contains seven items about opinions regarding EBP. Respondents answer these questions by checking a 10-cm line, ranging from 'extremely positive' to 'extremely negative' or from 'fully agree' to 'fully disagree', depending on the topic (see Table 2). The remaining questions on awareness and actual usage of EBP are dichotomous, multiple-choice, or open-ended, as becomes clear in the Results section.

The BARRIERS questionnaire contains 29 items that are answered on a four-point scale, ranging from 'no barrier' to 'large barrier', as well as a 'no opinion' option. In addition, the respondents can enter in open-ended questions which barriers and facilitators they find most important, as well as any other barriers or facilitators they perceive.

The described subgroup analyses were intended, in particular different medical specialties, doctors vs. nurses, and males vs. females, as these subgroups might differ in their appreciation and application of the EBP-principle, but this analysis would depend on a sufficient response rate. We have rephrased the text in lines 163-165 and 275-276.

Related, I was wondering whether >1.000 (exact number?) out of almost 5.000 HCP in the Maldives are registered with the Ministry, or a selection was made when inviting HCP to participate

Answer: Healthcare professionals were invited through the Maldivian Nurses Association, the EBP Champions team, and the CEOs of the Maldivian hospitals, in order to reach as many healthcare workers as possible. Also, the survey was promoted by word of mouth. We have added this information in lines 154-156.

Related, please provide IQR or SD in the tables where medians and means are provided.

Answer: We have added SDs to the mean values shown in Table 2. In Table 4, SDs are already provided.

The largest group of respondents were nurses (total >80% RN, CNS, enrolled nurse, nurse manager). These professionals often perceive more barriers in using EBP. Can you elaborate on the effect on outcomes in your study (i.e. more positive due to other HCP, or less progress due to this large group of nurses). Related, it was not always clear to me whether HCP in general or nurses, physicians or clinicians were referred to (i.e. line 130, line 164 and some of the questions: nurse or HCP?).

Answer: As we could not conduct a subgroup analysis of doctors vs. nurses, we cannot show any differential appreciation of barriers towards EBP between these two groups. 'HCPs' was used to indicate the whole group of respondents.

In the abstract, a semi quantitative design is mentioned, and in methods section a descriptive design. The latter seems more appropriate.

Answer: We used several questionnaires to appreciate different features of the EBP-principle. The McColl questionnaire yielded mainly quantitative data, the BARRIERS questionnaire more qualitative information. Hence, we labeled the study as semi-quantitative, descriptive survey.

VERSION 2 - REVIEW

**Reviewer**	**2**
**Name**	**Santos, D**
**Affiliation**	**Universidade de Coimbra**
**Date**	**05-Apr-2025**
**COI**	

The changes made have improved the manuscript. Thank you very much. I have no other comments to add.

**Reviewer**	**3**
**Name**	**Jongerden, Irene P.**
**Affiliation**	**Vrije Universiteit Amsterdam**
**Date**	**31-Mar-2025**
**COI**	

The authors have responded to my comments quite adequately, my main concerns have been addressed. The authors have improved the manuscript by their revisions. Minor details:

- suggestion to state that the practical suggestions are derived from the results as the authors state in their reply, rather than based (line 255)

- Aishath Hamid is author and acknowledged as EBP health champion. Author will do?

- HCPs' was used to indicate the whole group of respondents, that is clear, but who do the authors address with 'clinicians'?

VERSION 2 - AUTHOR RESPONSE

Response to Reviewer 3:
- We have stated in the text that the practical suggestions are based on the results of our survey (line 244).
- Mrs. Hamid is indeed one of the authors as she has contributed to the conduct of the survey and content of the manuscript. We have stated the 'EBP health champions team' as she is not affilated to a single institution.
- We have changed all instances of 'clinicians' into 'healthcare professionals' to avoid any confusion. These HCPs have been defined in the 'Participants' paragraph of the Methods section (line 96).
